# Benthic microbial biogeographic trends in the North Sea are shaped by an interplay of environmental drivers and bottom trawling effort

**DOI:** 10.1038/s43705-023-00336-3

**Published:** 2023-12-15

**Authors:** Guido Bonthond, Jan Beermann, Lars Gutow, Andreas Neumann, Francisco Rafael Barboza, Andrea Desiderato, Vera Fofonova, Stephanie B. Helber, Sahar Khodami, Casper Kraan, Hermann Neumann, Sven Rohde, Peter J. Schupp

**Affiliations:** 1 Institute for Chemistry and Biology of the Marine Environment (ICBM), Carl von Ossietzky University Oldenburg, Schleusenstrasse 1, 26382 Wilhelmshaven, Germany; 2 Alfred Wegener Institute Helmholtz Centre for Polar and Marine Research, Am Handelshafen 12, 27570 Bremerhaven, Germany; 3 Helmholtz Centre Hereon, 21502 Geesthacht, Germany; 4 Estonian Marine Institute, University of Tartu, Mäealuse 14, 12618 Tallinn, Estonia; 5 Department of Invertebrate Zoology and Hydrobiology, University of Lodz, 90-136 Lodz, Poland; 6 Senckenberg am Meer Wilhelmshaven, German Centre for Marine Biodiversity Research, Südstrand 44, 26382 Wilhelmshaven, Germany; 7 Thünen Institute of Sea Fisheries, Herwigstraße 31, 27572 Bremerhaven, Germany; 8 Helmholtz Institute for Functional Marine Biodiversity at the University of Oldenburg (HIFMB), Ammerländer Heerstrasse 231, D-26129 Oldenburg, Germany

**Keywords:** Microbial ecology, Macroecology, Biogeography

## Abstract

Microbial composition and diversity in marine sediments are shaped by environmental, biological, and anthropogenic processes operating at different scales. However, our understanding of benthic microbial biogeography remains limited. Here, we used 16S rDNA amplicon sequencing to characterize benthic microbiota in the North Sea from the top centimeter of 339 sediment samples. We utilized spatially explicit statistical models, to disentangle the effects of the different predictors, including bottom trawling intensity, a prevalent industrial fishing practice which heavily impacts benthic ecosystems. Fitted models demonstrate how the geographic interplay of different environmental and anthropogenic drivers shapes the diversity, structure and potential metabolism of benthic microbial communities. Sediment properties were the primary determinants, with diversity increasing with sediment permeability but also with mud content, highlighting different underlying processes. Additionally, diversity and structure varied with total organic matter content, temperature, bottom shear stress and bottom trawling. Changes in diversity associated with bottom trawling intensity were accompanied by shifts in predicted energy metabolism. Specifically, with increasing trawling intensity, we observed a transition toward more aerobic heterotrophic and less denitrifying predicted metabolism. Our findings provide first insights into benthic microbial biogeographic patterns on a large spatial scale and illustrate how anthropogenic activity such as bottom trawling may influence the distribution and abundances of microbes and potential metabolism at macroecological scales.

## Introduction

The biogeography of microbes is shaped by environmental, biological and anthropogenic processes that operate at different scales [1]. This includes the microbiota that colonize marine sediments in high cell densities [2, 3]. Marine sediments filter and accumulate organic and inorganic matter and play a crucial role in the biogeochemical cycling of carbon, nitrogen, sulfur and metals [4, 5]. Most of these processes are carried out by microbes, which are organized at the microscale into communities that are tightly attached to sediment grains [3]. At the scale of millimeters to centimeters, microbiota are metabolically sorted along vertical redox gradients [5, 6]. The aerobic heterotrophs that consume oxygen at the sediment surface are sequentially substituted underneath by anaerobes utilizing alternative electron acceptors, including nitrate, manganese/iron oxides, sulfate and finally carbon dioxide. Patterns in benthic microbial composition and diversity at macroecological scales (i.e., regional, continental and global; [7]), and how they arise from environmental drivers, have been studied less extensively [8] but correlate with sediment type [9], temperature [10], organic resource availability [11], primary production [12], macrofaunal bioturbation [13] and environmental disturbance [14].

While the benthic environment is subject to disturbance driven by currents, waves and storms [15], it also experiences anthropogenic disturbances [16], which potentially affect microbial biogeography. Bottom trawling, a prevalent fishing practice in shallow shelf sea regions, represents the most extensive anthropogenic disturbance to seabed habitats [17]. In the North Sea, more than 60% of the bottom surface is trawled once or more per year [18]. As large metal chains and heavy nets are dragged over the seafloor, the local environment is physically disturbed, resulting in a range of effects that act at different scales [19]. Trawling resuspends large amounts of sediment [20, 21], alters seabed morphology [22], destroys biogenic structures [23], and injures or kills benthic macrofauna [24–26]. Directly and indirectly, trawling may alter the benthic biogeochemistry by enhancing the oxygenation of buried organic matter, impacting the sequestration and remineralization of organic carbon [27–31]. It may also suppress benthic denitrification, and therewith potentially contribute to eutrophication [32–34]. As bioturbation and bioirrigation activity by macrofauna controls benthic oxygen and carbon fluxes at regional scales [35], bottom trawling may also affect benthic microbiota and microbial processes on larger temporal scales through its impact on macrofaunal populations, of which some species are highly susceptible to trawling [15, 26, 36, 37].

Based on this range of impacts on the benthic environment, its macrofauna and biogeochemistry, bottom trawling might also affect the composition and diversity of benthic microbiota at large scales. In this study, we present an analysis of regional scale patterns and putative determinants – including bottom trawling effort – of benthic microbial diversity and composition in the central to southeastern North Sea. This marine region is known as one of the most heavily trawled regions in the world, but trawling intensities are highly heterogeneous [18, 38]. Our aim was to identify the major determinants of the benthic microbial biogeography at the regional scale. We conducted 16S rRNA gene metabarcoding on 339 surface sediment samples from 149 sites across hundreds of kilometers, measured various environmental variables, obtained model-based estimates of bottom shear stress levels (natural disturbance) and high resolution bottom trawling intensities recorded by the Vessel Monitoring System (VMS). We utilized uni- and multivariate statistical models, that control for spatial autocorrelation, to disentangle these variables, each representing different hypotheses, and evaluate the direction and shape of their relationship. Specifically, we aimed to evaluate the hypothetical effects of bottom trawling intensity on regional scale microbial biogeography.

## Methods

### Sampling and sedimentological parameters

**Fig. 1:**
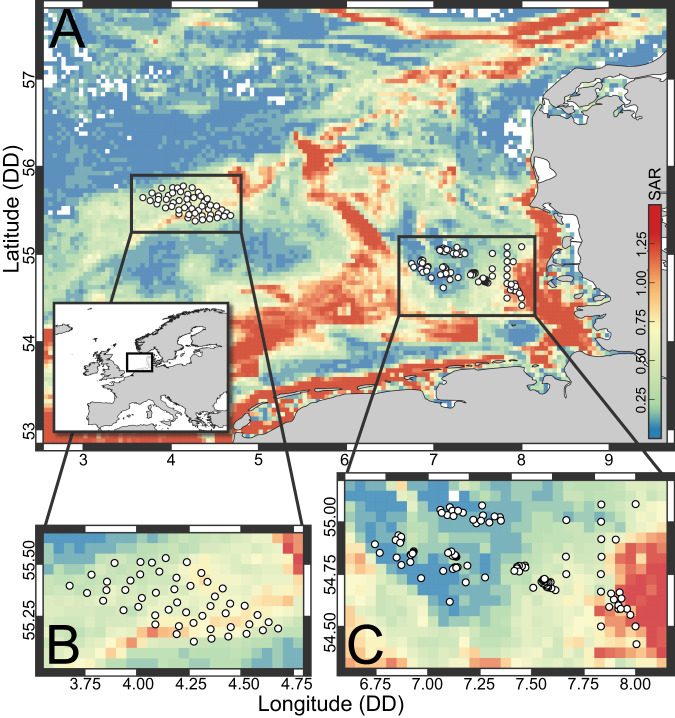
Geographic overview of the study area. **A** Map of the central and south-eastern North Sea with stations sampled with van Veen grabs. The background heatmap displays the bottom trawling intensity calculated as swept area ratio (SAR) per year (see the main text for details on SAR). Panels (**B**, **C**) show zoomed sections of the two areas sampled on two different expeditions (HE538 and HE562).

Sediment samples were taken with a 0.1 m^2^ van Veen grab (weight: 90 kg) during two scientific expeditions with the Research Vessel Heincke as part of an ecological long-term monitoring program (HE538, doi:10.2312/cr_he538 and HE562, doi:10.48433/cr_he562), in August 2019 and September 2020. In total, samples were collected from 150 stations in the southeastern North Sea, including 50 stations on the Dogger Bank (HE538) and 100 stations at the Sylt Outer Reef (HE562, Fig. 1). Sediment samples for DNA extraction were retrieved through a mesh lid on the top side of the grab to minimize disturbance and collected in 15 mL falcon tubes, taking the top centimeter only (HE538) or the top 1 to 10 centimeters as vertical core (HE562). In the latter case, three cores were taken approximately 10 cm apart in the same grab. Upon collection, tubes were stored at −20 °C on board, and afterwards transferred to the laboratory in cooling boxes, where they were stored again at −20 °C. For granulometry, a subsequent sample was taken using a core (diameter: 4.5 cm, penetration depth approximately 6 cm) from the same grab. The bottom temperature was determined from a separate grab with a standard thermometer, inserted to approximately 3 cm below the surface. Forty grams (wet weight) of the sub-sample were dried, weighed and combusted for 5 h at 500 °C. The total organic matter (TOM) content was calculated from the weight loss during combustion. The remaining sediment was fractionated in a sieve cascade with mesh sizes of 8000, 4000, 2000, 1000, 500, 250, 125 and 62.5 µm, corresponding to the Krumbein φ scale [39]. The mud (<62.5 µm), sand (>62.5 µm, <2000 µm) and pebble (>2000 µm) fractions were defined based on the percentage weight. For each sample, logistic regressions were fitted on the cumulative grain size distribution to estimate the median grain size by dividing the location and slope parameter multiplied by −1. The slope parameter was used as sorting coefficient.

### DNA extraction and library preparation

DNA was extracted from sub-samples of the top cm with the DNAeasy PowerSoil Pro kit (QIAGEN) following the manufacturer’s protocol. The 16SV3-V4 region was amplified with the primers 341F (S-D-Bact-0341-b-S-17) and 805R (S-D-Bact-0785-a-A-21, ref. [40]), and two amplicon libraries were prepared following Gohl et al. [41]. The first PCR was performed with the Phusion Green Hot Start II High-Fidelity PCR Master Mix (ThermoFisher) in volumes of 20 µL with 2 µL DNA template and 0.5 µM of each primer, an initial denaturation step of 98 °C for 3:00 min, 20 cycles of 98 °C for 0:30 min, 55 °C for 0:30 min and 72 °C for 0:30 min and a final elongation step of 72 °C for 5:00 min. To estimate relative amplicon concentrations in the final products before pooling the products into a library, the second PCR was conducted as qPCR in 20 µL volumes with the SsoAdvanced Universal SYBR Green Supermix (Biorad) applying the same program for 10 cycles. The qPCR products were then pooled in equimolar volumes based on the endpoint values, purified with a gel extraction step and sequenced on the Illumina MiSeq platform using the v3 600 cycle kit. Amplicon libraries included blanks from DNA extractions, negative PCR controls and mock communities (D6311, Zymo Research). Fastq files were demultiplexed and further processed and quality filtered with mothur v1.46.1[42], using the silva reference alignment v132 [43] for denoising and classification. Sequences were clustered into operational taxonomic units (OTUs) with the opticlust algorithm based on the 97% similarity criterion. Mitochondrial, chloroplast, eukaryotic and unclassified OTUs, singleton OTUs, and samples with less than 1000 reads were removed. Variation in sequencing depth among samples was accounted for by inclusion of the log transformed sequencing depth (LSD) as covariate in the statistical models. The raw demultiplexed amplicon reads were deposited in the SRA database (accession: PRJNA988469). Metagenomes with KEGG Ortholog (KO) annotations [44] were predicted with picrust2 v2.5.0 [45], using the default settings and excluding sequences without close relatives in the reference genomes.

### Statistical analysis

Eight variables were considered as potential drivers. As an estimate for contemporary bottom trawling intensity in the study area, we used subsurface (≥2 cm penetration of gear in the surface) fishing intensity from the OSPAR data & information management system [46] which is based on data from the vessel monitoring system (VMS). The data are expressed as swept area ratio (SAR) at a resolution of 0.05 squared decimal degrees, averaged over the years 2009–2017. SAR-values indicate the number of times an area is fished with bottom-touching gears per time-period. As a measure of natural disturbance, we used bottom shear stress (in N/m^2^). Spatial data on shear stress (maximum value) were derived from the barotropic FESOM-C setup with tidal forcing for the North Sea. FESOM-C is the coastal sub-unit of the global Finite-volumE Sea ice Ocean Model [47] and has been tested and verified in numerous idealized and realistic experiments for the North Sea [48–51]. The grid resolution varies from 30 to 100 m in nearshore areas to 1 km in deeper offshore areas. Tidal forcing was taken from the TPXO9 model [52]. The bottom friction coefficient varied spatially from 0.0025 to 0.003. The reference density field was averaged over the years 2019–2022 using salinity and potential bottom temperature for late August, which were taken from the NEMO setup for the North-West European shelf with a spatial resolution of 1.5 km [53]. Further, temperature (°C), pebble content (% weight), sand content (% weight), mud content (% weight), median grain size (mm) and TOM (proportion), were used as predictors. These variables were measured as described above. Pebble, sand and mud contents were square root transformed, TOM content was logit transformed and the median grain size was transformed to the Krumbein φ scale, by taking the negative *log*_2_ of the particle diameter in mm. Since all stations were below 10 m depth, light intensities were assumed to be negligible. Further, as shear stress is depth-dependent, declining with increasing depth due to the reduced influence of wind and wave activity, water depth was not considered in the modeling process.

Correlations among the potential predictors were assessed based on Spearman’s rank coefficients (Figure S1). In cases of strong correlations (|ρ| > 0.7), only one of the correlated variables was included in the modeling process [54].

Multivariate analyses were conducted with PERMANOVA [55] with the function *adonis2* from the R package vegan v2.6–4 and multivariate generalized linear models (mGLMs), assuming a negative binomial error distribution, using the *manyglm* function from the R package mvabund v4.2.1 [56]. To account for spatial autocorrelation, we followed the approach from Pelinson et al. [57] and computed Moran eigenvector maps (MEMs) with the R package adespatial v0.3–20. The positive MEMs were included as artificial spatial variables in PERMANOVAs and mGLMs. In addition, the PERMANOVAs and mGLMs included the LSD as covariate, to control for differences in total read counts among samples, and the predictors of interest (i.e., median grain size, mud content, TOM, temperature, bottom shear stress and trawling intensity). The marginal effect of each predictor on the overall OTU, genus and predicted KO composition was tested with PERMANOVA, using Aitchison distances and 9999 permutations. To consider the dependency among samples from the same grab, permutations were restricted to stations, by including station identity as a blocking factor. Based on the coefficients and standard errors from the mGLMs, we counted the number of OTUs and KOs with relative abundances varying with each predictor. Coefficients were considered significant when the theoretical 99% confidence region was entirely below (negative response) or above zero (positive response).

Non-metric multidimensional scaling (nMDS) based on Aitchison distances was conducted using the R package vegan v2.6–4. To exclude the effect of the sequencing depth prior to the scaling procedure, partial residuals from the mGLMs were used. Ordination vectors for the predictors of interest were computed with the *envfit* function from the same R package.

Alpha diversity was measured in effective numbers [58] of OTUs, genera and predicted KOs. As a measure of local scale beta diversity, we calculated Aitchison distances among cores from the same van Veen grab. Finally, we analyzed specific predicted functional groups related to energy metabolism, using the summed predicted ortholog counts from complete or partial KEGG pathway modules. This included the KOs for cytochrome c oxidase to represent aerobic respiration (M00155), nitrification (M00528), denitrification (M00529), dissimilatory nitrate reduction and dissimilatory nitrite reduction (M00530), thiosulfate sulfate oxidation (M00595), dissimilatory sulfate reduction (M00596), methanogenesis from CO_2_ (M00567) and methane oxidation (M00174). Details on the predicted KOs included in the modules are provided in Table S1.

Univariate responses were analyzed with generalized additive mixed models (GAMMs) using penalized cubic regression splines with the *gamm* function from the R package mgcv v1.8–41 [59]. First a global model was fitted, including all predictors (retained after the correlation analysis) as smooths with a maximum number of 3 degrees of freedom for each term. Station identity was included as random intercept to represent non-independence among samples from the same grab and the LSD was used as a covariate in the model to correct for the sequencing depth. To account for spatial autocorrelation, the models contained an exponential covariance structure, using the geospatial coordinates. Within grabs, the latitudinal coordinates of the outer samples were adjusted with ± 10^−6^ latitudinal decimal degrees to approximate the 10 cm distance between samples within a grab. Then, we compared the global model with the simpler models generated from all possible combinations of fixed effects, but always including the random effect and covariance structure, and selected the best model based on the AIC_c_ criterion. The relative importance (RI) of retained predictors was calculated as the sum of AIC_cw_ of all models with a ∆AIC_c_ ≤ 4 [60].

## Results

**Fig. 2:**
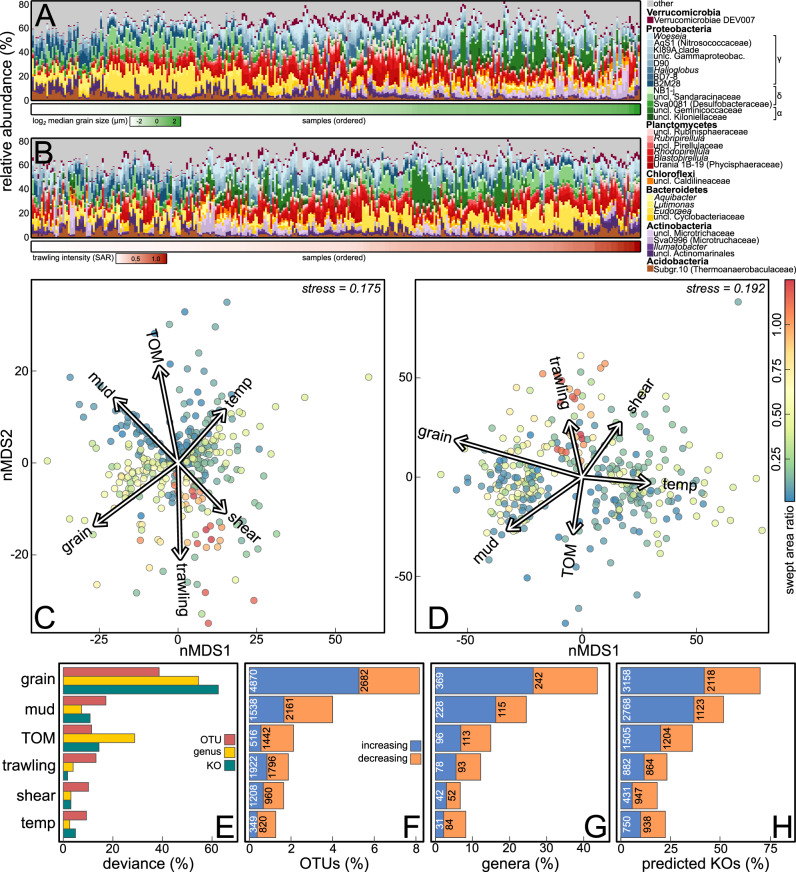
Composition and patterns in benthic microbiota. Stacked bar plots of the 25 most abundant prokaryotic genera across all samples, (**A**) sorted by median grain size and (**B**) by swept area ratio. nMDS plots based on Aitchison distances displaying compositional dissimilarities of the microbial communities after correcting for the sequencing depth, based on (**C**) OTUs and (**D**) predicted KOs. The environmental variables were ordinated on the nMDS plots and are displayed as vectors. **E** The deviance explained by each term as percentage of the overall deviance explained by the model. The number of (**F**) OTUs, (**G**) genera and (**H**) predicted KOs, which differ in abundance with environmental predictors.

The quality filtered dataset counted 91,860 bacterial and 612 archaeal OTUs across 339 samples from 149 stations. *Woeseia* (Gammaproteobacteria) was the dominant taxon in most samples, followed by unclassified Sandaracinaceae (Deltaproteobacteria), unclassified Actinomarinales (Actinobacteria), Sva0996-marine-group (Microtrichaceae, Actinobacteria), *Rhodopirellula* and *Blastopirellula* (Planctomycetes). Of these taxa, Sva0996-marine-group and the unclassified Sandaracinaceae (Deltaproteobacteria) were dominant in muddy sediments, whereas *Woeseia* (Gammaproteobacteria) and *Blastopirellula* (Planctomycetes) dominated in samples with high median grain size (Fig. 2A, Table S2). Communities from areas with no or low trawling intensity tended to be dominated by unclassified Gammaproteobacteria and *Lutimonas* (Bacteroidetes) whereas heavily trawled sites tended to be dominated by *Woeseia* and unclassified Sandaracinaceae (Gamma- and Deltaproteobacteria, respectively; Fig. 2B).

In total 7550 KEGG orthologs (KOs) were predicted with picrust2. 896 OTUs (0.96% of all OTUs) from the OTU table had nearest-neighbor sequence index (NSTI) values > 2 (the default threshold) and were excluded prior to the prediction process. The weighted NSTI per sample varied from 0.172 to 0.266 (median 0.207), within the range of ocean and soil microbiota datasets from which predictions have been obtained with reasonable precision [45].

### Correlation analysis

Strong correlations (|*ρ*| > 0.7) were detected among pebble content, sand content, median grain size and sediment sorting. Mud content was correlated weakly with the other sediment variables (|*ρ*| < 0.5). Therefore, the model selection was conducted with the median grain size to represent the coarser sediment fractions and include mud content as separate variable. Apart from a moderate correlation (0.5 < |*ρ*| < 0.7) between TOM content and trawling intensity (*ρ* = −0.51) all other correlations were weak (|*ρ*| < 0.5, Figure S1).

### Community composition

Community composition was spatially autocorrelated, as indicated by two positive MEMs, which were used as artificial spatial variables in the PERMANOVAs and mGLMs to account for spatial autocorrelation. All included predictors had significant marginal effects on OTU and predicted KO composition (p < 0.05, Table S3). At the genus level, all variables except TOM content and shear stress were significant. These effects were visible in nMDS plots (Fig. 2C, D). For OTU, genus and predicted KO composition, the median grain size was most important, explaining 38.7%, 54.6% and 62.5%, respectively, of the overall explained deviance. Mud content was the next most important predictor for OTU composition (17.2%) and TOM content for genus and predicted KO composition (28.8% and 14.4%). Bottom trawling intensity was less important but explained 13.2%, 4.0% and 4.2% of the overall deviance in OTU, genus and predicted KO composition, respectively (Fig. 2E). The median grain size also yielded the most differentially abundant OTUs, genera and predicted KOs (7,552 OTUs, 611 genera, 5,276 KOs). In total 1,731 OTUs, 171 genera and 1,746 predicted KOs varied with bottom trawling intensity (Fig. 2F–H, see Figure S2 for more details on differentially abundant genera).

### Diversity

**Fig. 3:**
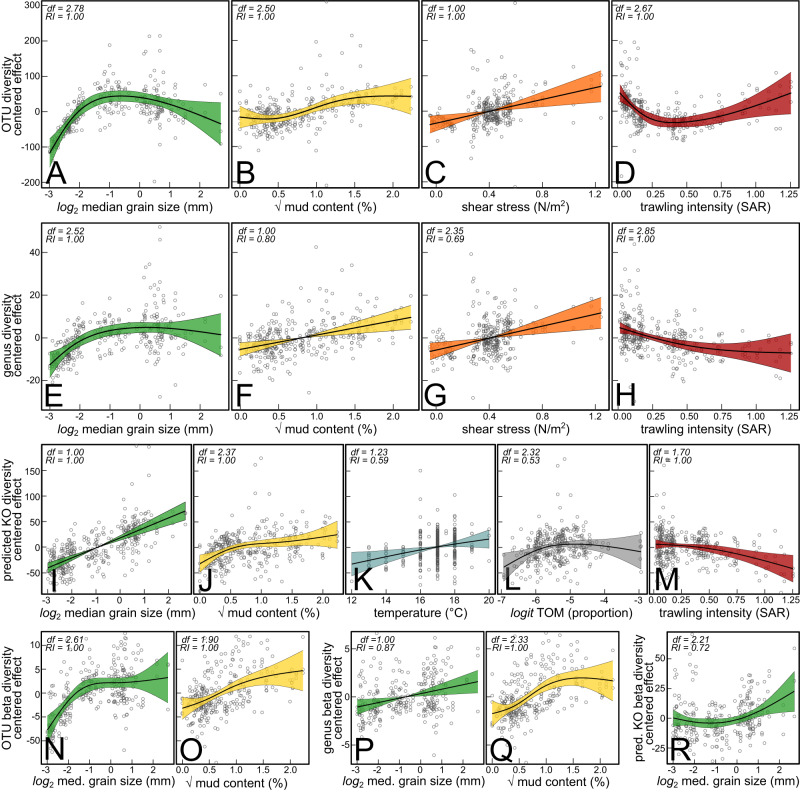
Trends in benthic microbial diversity. Partial effects estimated by generalized additive mixed models (GAMM) fitted on (**A**–**D**) OTU alpha diversity, (**E**–**H**) genus level alpha diversity, (**I**–**M**) predicted KO alpha diversity, (**N**–**O**) OTU beta diversity and (**P**, **Q**) genus level beta diversity and (**R**) predicted KO beta diversity, in response to the environmental predictors retained after model selection (see main text for further details). The vertical axes are centered and expressed in the scale of the response. The estimated degrees of freedom (*df*) of each smooth and the relative importance (RI) of each term are indicated in the top left corner. Shaded areas indicate the 95% confidence regions.

Alpha diversity (effective OTU and genus numbers) was best explained by the median grain size, mud content, temperature, bottom shear stress and trawling intensity (Fig. 3A–H, Table S4-5). OTU and genus diversity increased with the median grain size (Fig. 3A, E) and mud content (Fig. 3B, F). Both at the OTU and genus level, diversity increased also with shear stress (Fig. 3C, G). In response to trawling, OTU diversity dropped rapidly at relatively low trawling intensities (SAR < 0.25) but increased again gradually at higher trawling rates (SAR > 0.5) (Fig. 3D). Genus diversity decreased with bottom trawling (Fig. 3H). For predicted functional diversity, the model selection procedure yielded median grain size, mud content, temperature, TOM content and trawling intensity as informative predictors. Predicted functional diversity increased with median grain size, mud content and temperature (Fig. 3I–L) and decreased with trawling intensity (Fig. 3M).

OTU and genus beta diversity were best explained by median grain size and mud content, both showing increasing trends with median grain size (Fig. 3N–Q). Median grain size was the only informative predictor for predicted functional beta diversity, with which it increased (Fig. 3R).

### Predicted metabolic groups

**Fig. 4:**
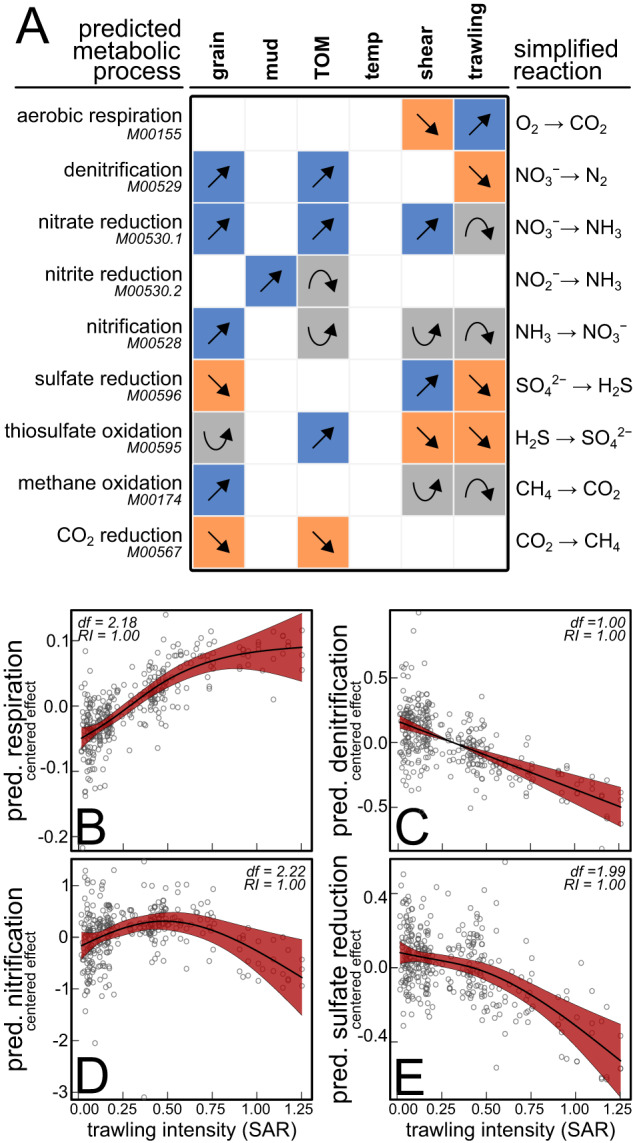
Potential metabolism of benthic microbiota **A** Simplified trends estimated by GAMMs fitted on several predicted metabolic groups, based on KEGG gene modules (see main text for details). Partial effects estimated by GAMMs for bottom trawling only on predicted (**B**) aerobic respiration, (**C**) denitrification, (**D**) nitrification and (**E**) sulfate reduction. The vertical axes are centered and expressed on the scale of the response (which was expressed as the natural logarithm of the sum of predicted KOs in the module). The estimated degrees of freedom (df) of each smooth and the relative importance (RI) of each term are indicated in the top left corner. Shaded areas show the 95% confidence regions.

Median grain size was retained in all models for the predicted metabolic groups except for aerobic respiration and dissimilatory nitrite reduction (Fig. 4A). Mud content was an informative predictor for dissimilatory nitrite reduction. TOM content explained changes in predicted aerobic respiration, denitrification, nitrate reduction, nitrite reduction, thiosulfate oxidation and CO_2_ reduction. Further, bottom shear stress was informative for predicted aerobic respiration, nitrate reduction, nitrification, dissimilatory sulfate reduction, thiosulfate reduction and methane oxidation and bottom trawling intensity for aerobic respiration (Fig. 4B), nitrification (Fig. 4C), methane oxidation, denitrification (Fig. 4D), dissimilatory nitrate reduction, sulfate reduction (Fig. 4B) and thiosulfate oxidation.

## Discussion

We characterized the microbial biogeography of the top sediment layer at a regional scale (i.e., across hundreds of kilometers) in the central to southeastern North Sea. All environmental variables included in our analyses partially explained at least some of the taxonomic and/or functional responses in benthic microbiota. This is in line with previous studies, which identified sediment characteristics, bottom temperature, and organic matter content as important variables shaping microbiota at larger scales [3, 9, 11, 61]. Microbial communities were spatially autocorrelated, indicating the effect of unmeasured variables and/or historical contingencies. After accounting for environmental effects and spatial autocorrelation, we also found patterns in composition, diversity and predicted functions associated with bottom shear stress and bottom trawling intensity. Therewith, our work adds to previous studies demonstrating the impact of bottom trawling on microbial biogeochemical processes [32, 34, 62, 63] and provides evidence that bottom trawling may influence benthic microbial biogeography and potential metabolism.

### Granular properties

Sediment properties, represented by median grain size and mud content, were the primary determinants of benthic microbial diversity and composition. The median grain size and mud content explained different parts of the overall variation, indicating that these variables mirror different underlying processes possibly acting on different scales. The median grain size is a good proxy for sediment permeability [64, 65], and thus of porewater advection rates across sediments layers (i.e., centimeters), whereas mud particles may constrain advection at much smaller scales (i.e. micrometers) by forming cohesive aggregates wherein diffusion is the dominant process for solute transport. Mud aggregates could promote whole sediment diversity through the formation of microenvironments with functionally different communities, a process which is also known from soils [66]. Advection may promote alpha diversity rather through ecological processes such as dispersal and community coalescence between sediment layers and the water column. This may not only explain the observed patterns in alpha diversity, but also in predicted functional beta diversity, for which only the median grain size but not mud content was a good predictor. Whereas the occurrence of mud aggregates likely has a specific effect at microscales, advection could also enhance the stochastic occurrence of microbes from the water column or deeper sediment layers, which may increase within-grab beta diversity. Previous studies have reported a negative correlation between permeability and microbial diversity but attributed this to the typically higher organic content in muddy sediments [9, 67]. Our analyses disentangle the effects of median grain size, mud content and TOM content by estimating partial effects of each predictor (i.e., how diversity varies with median grain size when levels of mud and TOM content are kept constant). Based on this, we suggest that the correlations reported in Franco et al. [67] and Probandt et al. [9] are most likely explained by confounding mud content, TOM content or both, rather than by permeability itself.

### Total organic matter content

Globally, organic matter content explains microbial community composition in marine sediments [11] as well as microbial biomass and diversity in terrestrial soils [68]. Our study in the North Sea confirms that benthic microbial composition and predicted functional diversity vary with TOM content. The increasing to unimodal trend in functional diversity agrees with macroecological theory which predicts that increased resource availability promotes diversity up to an optimum, beyond which biological processes (e.g., competition) favor specific functional groups at the costs of others [69].

We also observed a relative increase in several predicted anaerobic groups (e.g., denitrification, thiosulfate oxidation, Fig. 4). This may mirror a transition from organic substrate limitation to oxygen limitation as resource availability increases, which could facilitate the proliferation of anaerobic heterotrophs, utilizing alternative electron acceptors. In the North Sea, high TOM content typically occurs in areas with low levels of near-bed turbulence and low permeability [35]. Here, absolute levels of oxygen consumption are relatively high [70]. Under these combined conditions of high TOM content and low oxygen supply through decreased advection, oxygen may be particularly depleted near the sediment surface or in microenvironments such as dead-end pores and grain crevices, which could further boost anaerobic metabolism in the surface layer.

### Bottom temperature

The influence of temperature on the microbial composition and diversity is well established [71–74] and our results corroborate that bottom temperature is an important predictor for benthic microbial composition and functional diversity. Thermal stress is often linked to microbial beta diversity in holobionts [74–77] according to the Anna Karenina Principle, which predicts beta diversity to increase due to a shift from deterministic to more stochastic processes acting on communities [75]. In a recent study on marine sediments, temperature was also identified as major driver of local scale beta diversity [78]. Within the thermal range of this study (12 °C to 20 °C), we could not confirm a temperature-beta diversity relationship. While temperature was associated with predicted functional composition and diversity, it did not explain changes in the predicted groups related to energy metabolism (Fig. 4A), suggesting it rather affects traits not directly related with energy metabolism.

### Bottom shear stress

How microbial diversity varies along gradients of physical disturbance has received limited attention, but some studies found that diversity declines with disturbance in soils [79] or follows a unimodal trend in coastal marine sediments in agreement with the intermediate disturbance hypothesis [14]. Within the range of disturbance levels estimated for the present study, we found a linear trend, with OTU and genus diversity increasing toward higher levels of disturbance. In surface sediments, near-bottom turbulence enhances pore water advection rates, but also promotes other modes of transport such as sediment erosion and mobile bedforms [65]. For example, the frequent resuspension of the surface sediment by mobile bedforms is constantly mixing individual grains into new redox zones and may prevent the local microbial community of a particular grain to complete succession to specific redox conditions. In this way, high bottom shear stress levels could increase alpha diversity by preventing communities to reach successional stages where diversity is restricted by strong competition. Notably, bottom shear stress was not an informative predictor for local beta diversity. This may seem counterintuitive to the idea that higher advection rates (i.e., higher median grain size) promote beta diversity by increasing random occurrence of microbes from the water column or deeper layers. However, while bottom shear stress indeed increases porewater advection rates, it also mixes and resuspends the sediment itself, which may decrease within-grab beta diversity at the same time by reducing substrate heterogeneity existing at the scale of centimeters or more (e.g., from macrofaunal bioturbation).

### Bottom trawling

VMS based SAR estimates of fishing intensity are aggregated in space and time. Therefore, SAR values linked to datapoints do not necessarily reflect actual trawling events, as a given station that has never been trawled, may lay in a heavily trawled grid cell [80]. Moreover, SAR does not distinguish short- and long-term effects, nor effects operating within and outside of trawling tracks and thus represent rather a probability that a geospatial position is in some way impacted by trawling. Consequently, this limits the ability to detect trawling effects and poses the need for high spatial replication to evaluate bottom trawling impact. In spite of this, benthic macrofaunal diversity and biomass have been found to decrease with SAR [23, 26, 81]. Here, taxonomic and predicted functional microbial diversity followed similar trends in response to trawling intensities. The rapid decline in OTU diversity at low trawling intensity (from 0 to 0.25 SAR) toward a minimum may indicate a depletion effect, with the first trawling pressure having the highest impact and the effect of every subsequent trawl being proportional to the previous one [82]. The more linear decrease in genus level and functional diversity suggests that the relatively high loss in OTU diversity at low trawling rates and increase at higher trawling rates primarily concerns closely related and functionally redundant OTUs within only few groups, while diversity at higher ranks responded more slowly and continues to decline.

The effect of bottom trawling on macrofaunal communities may overlap with the effects of natural mechanical disturbance [15, 37]. Based on our findings, this may partially apply to microbial composition, as bottom trawling and bottom shear stress were ordinated in similar directions in the nMDS (Fig. 2C, D). However, in terms of taxonomic diversity, trawling and shear stress yielded contrasting trends. In contrast to bottom shear stress, which was only informative for taxonomic diversity, bottom trawling was also associated with decreasing predicted functional diversity and yielded different trends in predicted metabolic groups, which suggests differential effects of the two types of disturbance, potentially reaching down to the functional level. While the nature of disturbance is similar in some respects (e.g., sediment resuspension and mixing of the upper sediment layers), bottom trawling has different effects on the seabed morphology [22], and is more lethal to certain benthic macrofaunal species [15, 37].

The trends related to energy metabolism uncovered in this study are based on functional profiles inferred from amplicon data and should thus be interpreted with caution as they only represent a prediction or potential of the functional potential. Nevertheless, the relative increase in predicted aerobic energy metabolism (i.e., aerobic respiration, nitrification) and decrease in predicted anaerobic metabolic groups (i.e., denitrification, dissimilatory sulfate reduction), could mirror a trawling induced shift from anaerobic to aerobic heterotrophy within microbial communities. Microbial heterotrophy plays an important role in the storage and remineralization of organic carbon in marine sediments [4, 5]. Trawling may impede carbon storage or induce underwater carbon dioxide emissions by increasing aerobic microbial respiration [30], although in which quantities this occurs is subject of debate [83]. Aerobic heterotrophy can be enhanced in different ways, including degradation of seabed morphology [18, 22], re-exposure of buried organic matter to aerobic conditions [32, 62, 63], or by impacting macrofauna [13, 31]. While the redistribution of labile organic matter from deeper layers may fuel aerobic microbial heterotrophy [29, 62], the putative trawling impact on burrowing macrofauna could further enhance aerobic microbial respiration, as benthic invertebrates consume most of the oxygen in the top sediment layer [5] and transport labile organic matter to deeper anaerobic layers, allowing organic matter to escape aerobic microbial decomposition [13, 31, 35].

A concurrent relative decrease in predicted denitrification, may indicate a shift in metabolism related to nitrogen cycling to be associated with trawling. Also this biogeographic trend aligns with biogeochemical patterns detected in previous studies that evaluated bottom trawling impact and reported reduced denitrification and increased sediment nitrate concentrations [32, 33, 63]. Denitrification, which plays a major role in the removal of bioavailable nitrogen from marine systems such as the North Sea [84], is maximized within marine sediments by the three-dimensional complexity of the redox structure which is formed by burrowing macrofauna [13, 33]. Thus, both the mechanical trawling impact and the putative effects on macrofaunal communities, may explain the shift in predicted nitrogen metabolism within benthic microbiota.

While the negative effects of bottom trawling on benthic macrofaunal diversity and biomass are well established for various systems, including the North Sea [23, 26, 36, 37], the susceptibility of macrofaunal communities to trawling is area specific and likely varies at different spatial scales [15]. Therefore, the effects on the macrofaunal communities are not simply a function of SAR values and our results emphasize the need for future study to evaluate how the biogeography of benthic microbiota and macrofauna covary in the context of bottom trawling.

## Conclusions

Our models successfully explained regional scale environmental patterns in benthic microbial biogeography. Generally, sediment characteristics were identified as the most important determinants of structure and functioning. We posit that both alpha and beta diversity are shaped by processes operating at different spatial scales: At the micron scale, mud content enhances whole sediment diversity by the formation of microscale environments. At the scale of centimeters, pore water advection may promote alpha and beta diversity by facilitating the exchange of microbial cells across sediment layers. Finally, operating at the scale of meters or more, bottom shear stress may enhance alpha diversity in parallel, by promoting porewater exchange. After accounting for environmental predictors, bottom shear stress and spatial autocorrelation, microbiota varied also with bottom trawling, revealing a decrease in alpha and predicted functional diversity with increasing trawling intensity. At low taxonomic ranks (i.e. OTU level), diversity increased again at higher trawling rates, but this was not followed by genus and predicted functional diversity, which gradually declined further. The effects of bottom trawling on community composition were partially similar to those of bottom shear stress, potentially indicating overlapping effects of the two types of physical disturbance. However, they were associated with different changes in diversity and in predicted functions. Specifically, two noteworthy patterns in potential energy metabolism emerged, including a relative shift towards more potential aerobic metabolism and toward less potential denitrification. While our data cannot be translated to chemical fluxes, and may not necessarily indicate changes in biogeochemical cycling, they do reveal notable biogeographic trends at a regional scale, which have not been documented before. Ultimately, the results of this study emphasize that the microbial biogeographic consequences of bottom trawling activity at the global scale merit further research.

### Supplementary information

Supplementary information

## Data Availability

The raw de-multiplexed V3-V4-16S gene amplicon reads and associated metadata are available from the SRA database under the Bioproject accession number PRJNA988469. Data and R-scripts for analyses are available on GitHub at https://github.com/gbonthond/srf_benthic_microbial_biogeography.

## References

[1] Martiny JBH, Bohannan BJM, Brown JH, Colwell RK, Fuhrman JA, Green JL (2006). Microbial biogeography: putting microorganisms on the map. Nat Rev Microbiol.

[2] Musat N, Werner U, Knittel K, Kolb S, Dodenhof T, van Beusekom JEE (2006). Microbial community structure of sandy intertidal sediments in the North Sea, Sylt-Rømø Basin, Wadden Sea. Syst Appl Microbiol.

[3] Probandt D, Eickhorst T, Ellrott A, Amann R, Knittel K (2018). Microbial life on a sand grain: from bulk sediment to single grains. ISME J.

[4] Middelburg JJ (2018). Reviews and syntheses: to the bottom of carbon processing at the seafloor. Biogeosciences.

[5] Jørgensen BB, Wenzhöfer F, Egger M, Glud RN (2022). Sediment oxygen consumption: Role in the global marine carbon cycle. Earth-Sci Rev.

[6] Chen J, Hanke A, Tegetmeyer HE, Kattelmann I, Sharma R, Hamann E (2017). Impacts of chemical gradients on microbial community structure. ISME J.

[7] Gaston KJ, Blackburn TM (Eds.). (2000). Pattern and Process in Macroecology. 1st edn. Wiley.

[8] Shade A, Dunn RR, Blowes SA, Keil P, Bohannan BJM, Herrmann M (2018). Macroecology to unite all life, large and small. Trends Ecol Evolut.

[9] Probandt D, Knittel K, Tegetmeyer HE, Ahmerkamp S, Holtappels M, Amann R (2017). Permeability shapes bacterial communities in sublittoral surface sediments: Permeability shapes benthic bacterial communities. Environ Microbiol.

[10] Hicks N, Liu X, Gregory R, Kenny J, Lucaci A, Lenzi L (2018). Temperature driven changes in benthic bacterial diversity influences biogeochemical cycling in coastal sediments. Front. Microbiol..

[11] Hoshino T, Doi H, Uramoto G-I, Wörmer L, Adhikari RR, Xiao N (2020). Global diversity of microbial communities in marine sediment. Proc Natl Acad Sci USA..

[12] Zinger L, Amaral-Zettler LA, Fuhrman JA, Horner-Devine MC, Huse SM, Welch DBM (2011). Global patterns of bacterial beta-diversity in seafloor and seawater ecosystems. PLoS ONE.

[13] Laverock B, Tait K, Gilbert JA, Osborn AM, Widdicombe S (2014). Impacts of bioturbation on temporal variation in bacterial and archaeal nitrogen‐cycling gene abundance in coastal sediments. Environ Microbiol Rep.

[14] Galand PE, Lucas S, Fagervold SK, Peru E, Pruski AM, Vétion G, et al. Disturbance increases microbial community diversity and production in marine sediments. Front Microbiol. 2016;7:1950. 10.3389/fmicb.2016.01950 PMC5133735 27994581

[15] van Denderen P, Bolam S, Hiddink J, Jennings S, Kenny A, Rijnsdorp A (2015). Similar effects of bottom trawling and natural disturbance on composition and function of benthic communities across habitats. Mar Ecol Prog Ser..

[16] Halpern BS, Walbridge S, Selkoe KA, Kappel CV, Micheli F, D’Agrosa C (2008). A global map of human impact on marine ecosystems. Science.

[17] Kaiser MJ, Collie JS, Hall SJ, Jennings S, Poiner IR (2002). Modification of marine habitats by trawling activities: prognosis and solutions. Fish Fisheries.

[18] Eigaard OR, Bastardie F, Hintzen NT, Buhl-Mortensen L, Buhl-Mortensen P, Catarino R (2017). The footprint of bottom trawling in European waters: distribution, intensity, and seabed integrity. ICES J Marine Sci.

[19] Piet GJ, Quirijns FJ (2009). The importance of scale for fishing impact estimations. Can J Fish Aquat Sci..

[20] Rijnsdorp AD, Depestele J, Molenaar P, Eigaard OR, Ivanović A, O’Neill FG (2021). Sediment mobilization by bottom trawls: a model approach applied to the Dutch North Sea beam trawl fishery. ICES J Marine Sci.

[21] Breimann SA, O’Neill FG, Summerbell K, Mayor DJ (2022). Quantifying the resuspension of nutrients and sediment by demersal trawling. Continental Shelf Res.

[22] Puig P, Canals M, Company JB, Martín J, Amblas D, Lastras G (2012). Ploughing the deep sea floor. Nature.

[23] Tillin H, Hiddink J, Jennings S, Kaiser M (2006). Chronic bottom trawling alters the functional composition of benthic invertebrate communities on a sea-basin scale. Mar Ecol Prog Ser..

[24] Bergman M, Hup M (1992). Direct effects of beamtrawling on macrofauna in a sandy sediment in the southern North Sea. ICES J Marine Sci.

[25] Bolam SG, Coggan RC, Eggleton J, Diesing M, Stephens D (2014). Sensitivity of macrobenthic secondary production to trawling in the English sector of the Greater North Sea: A biological trait approach. J Sea Res.

[26] Hiddink JG, Jennings S, Sciberras M, Szostek CL, Hughes KM, Ellis N (2017). Global analysis of depletion and recovery of seabed biota after bottom trawling disturbance. Proc Natl Acad Sci USA.

[27] Falcão M, Gaspar MB, Caetano M, Santos MN, Vale C (2003). Short-term environmental impact of clam dredging in coastal waters (south of Portugal): chemical disturbance and subsequent recovery of seabed. Marine Environ Res.

[28] Warnken KW, Gill GA, Dellapenna TM, Lehman RD, Harper DE, Allison MA (2003). The effects of shrimp trawling on sediment oxygen consumption and the fluxes of trace metals and nutrients from estuarine sediments. Estuarine Coastal Shelf Sci.

[29] Morys C, Brüchert V, Bradshaw C (2021). Impacts of bottom trawling on benthic biogeochemistry in muddy sediments: Removal of surface sediment using an experimental field study. Marine Environ Res.

[30] Sala E, Mayorga J, Bradley D, Cabral RB, Atwood TB, Auber A (2021). Protecting the global ocean for biodiversity, food and climate. Nature.

[31] Epstein G, Middelburg JJ, Hawkins JP, Norris CR, Roberts CM (2022). The impact of mobile demersal fishing on carbon storage in seabed sediments. Global Change Biol.

[32] De Borger E, Tiano J, Braeckman U, Rijnsdorp AD, Soetaert K (2021). Impact of bottom trawling on sediment biogeochemistry: a modelling approach. Biogeosciences.

[33] Ferguson AJP, Oakes J, Eyre BD (2020). Bottom trawling reduces benthic denitrification and has the potential to influence the global nitrogen cycle. Limnol Oceanogr Lett.

[34] Tiano JC, Depestele J, Van Hoey G, Fernandes J, van Rijswijk P, Soetaert K (2022). Trawling effects on biogeochemical processes are mediated by fauna in high-energy biogenic-reef-inhabited coastal sediments. Biogeosciences.

[35] Neumann A, van Beusekom JEE, Eisele A, Emeis K, Friedrich J, Kröncke I (2021). Macrofauna as a major driver of bentho‐pelagic exchange in the southern North Sea. Limnology Oceanogr.

[36] Reiss H, Greenstreet S, Sieben K, Ehrich S, Piet G, Quirijns F (2009). Effects of fishing disturbance on benthic communities and secondary production within an intensively fished area. Mar Ecol Prog Ser..

[37] Rijnsdorp AD, Bolam SG, Garcia C, Hiddink JG, Hintzen NT, van Denderen PD (2018). Estimating sensitivity of seabed habitats to disturbance by bottom trawling based on the longevity of benthic fauna. Ecol Appl.

[38] Kroodsma DA, Mayorga J, Hochberg T, Miller NA, Boerder K, Ferretti F (2018). Tracking the global footprint of fisheries. Science.

[39] Krumbein WC (1934). Size frequency distributions of sediments. J Sedimentary Res.

[40] Klindworth A, Pruesse E, Schweer T, Peplies J, Quast C, Horn M (2013). Evaluation of general 16S ribosomal RNA gene PCR primers for classical and next-generation sequencing-based diversity studies. Nucleic Acids Res.

[41] Gohl DM, Vangay P, Garbe J, MacLean A, Hauge A, Becker A (2016). Systematic improvement of amplicon marker gene methods for increased accuracy in microbiome studies. Nat Biotechnol.

[42] Schloss PD, Westcott SL, Ryabin T, Hall JR, Hartmann M, Hollister EB (2009). Introducing mothur: open-source, platform-independent, community-supported software for describing and comparing microbial communities. Appl Environ Microbiol.

[43] Quast C, Pruesse E, Yilmaz P, Gerken J, Schweer T, Yarza P (2013). The SILVA ribosomal RNA gene database project: improved data processing and web-based tools. Nucleic Acids Res.

[44] Kanehisa M, Goto S, Sato Y, Kawashima M, Furumichi M, Tanabe M (2014). Data, information, knowledge and principle: back to metabolism in KEGG. Nucl Acids Res.

[45] Douglas GM, Maffei VJ, Zaneveld JR, Yurgel SN, Brown JR, Taylor CM (2020). PICRUSt2 for prediction of metagenome functions. Nat Biotechnol.

[46] OSPAR. OSPAR Bottom Fishing Intensity - Surface & Subsurface. https://odims.ospar.org/en/maps/map-bottom-fishing-i_-surface-subsurface_khexe/ 2017.

[47] Androsov A, Fofonova V, Kuznetsov I, Danilov S, Rakowsky N, Harig S (2019). FESOM-C v.2: coastal dynamics on hybrid unstructured meshes. Geosci Model Dev..

[48] Fofonova V, Androsov A, Sander L, Kuznetsov I, Amorim F, Hass HC (2019). Non-linear aspects of the tidal dynamics in the Sylt-Rømø Bight, south-eastern North Sea. Ocean Sci.

[49] Fofonova V, Kärnä T, Klingbeil K, Androsov A, Kuznetsov I, Sidorenko D (2021). Plume spreading test case for coastal ocean models. Geosci Model Dev..

[50] Kuznetsov I, Androsov A, Fofonova V, Danilov S, Rakowsky N, Harig S (2020). Evaluation and application of newly designed finite volume coastal model FESOM-C, effect of variable resolution in the Southeastern North Sea. Water.

[51] Sprong PAA, Fofonova V, Wiltshire KH, Neuhaus S, Ludwichowski KU, Käse L (2020). Spatial dynamics of eukaryotic microbial communities in the German Bight. J Sea Res.

[52] Egbert GD, Erofeeva SY (2002). Efficient inverse modeling of barotropic ocean tides. J Atmos Oceanic Technol.

[53] Copernicus Marine Service. (2018). Atlantic - European North West Shelf - Ocean Physics Analysis and Forecast.

[54] Dormann CF, Elith J, Bacher S, Buchmann C, Carl G, Carré G (2013). Collinearity: a review of methods to deal with it and a simulation study evaluating their performance. Ecography.

[55] Anderson MJ (2001). A new method for non-parametric multivariate analysis of variance: NON-PARAMETRIC MANOVA FOR ECOLOGY. Austral Ecol.

[56] Wang Y, Naumann U, Wright ST, Warton DI (2012). mvabund - an R package for model-based analysis of multivariate abundance data: The mvabund R package. Methods Ecol Evolut.

[57] Pelinson RM, Leibold MA, Schiesari L. Top predator introduction changes the effects of spatial isolation on freshwater community structure. Ecology. 2021;102: e03500. 10.1002/ecy.3500 34314027

[58] Jost L (2006). Entropy and diversity. Oikos.

[59] Wood SN (2011). Fast stable restricted maximum likelihood and marginal likelihood estimation of semiparametric generalized linear models: Estimation of Semiparametric Generalized Linear Models. J R Stat Soc: Series B (Statistical Methodology).

[60] Burnham KP, Anderson DR (Eds.). (2004). Model Selection and Multimodel Inference. Springer New York, New York, NY.

[61] Wang L, Zheng B, Nan B, Hu P (2014). Diversity of bacterial community and detection of nirS- and nirK-encoding denitrifying bacteria in sandy intertidal sediments along Laizhou Bay of Bohai Sea, China. Marine Pollut Bull.

[62] van de Velde S, Van Lancker V, Hidalgo-Martinez S, Berelson WM, Meysman FJR (2018). Anthropogenic disturbance keeps the coastal seafloor biogeochemistry in a transient state. Sci Rep.

[63] Tiano JC, Witbaard R, Bergman MJN, van Rijswijk P, Tramper A, van Oevelen D (2019). Acute impacts of bottom trawl gears on benthic metabolism and nutrient cycling. ICES Mar Sci Symp..

[64] Gangi AF (1985). Permeability of unconsolidated sands and porous rocks. J Geophys Res..

[65] Ahmerkamp S, Winter C, Krämer K, Beer Dde, Janssen F, Friedrich J (2017). Regulation of benthic oxygen fluxes in permeable sediments of the coastal ocean: Regulation of benthic oxygen fluxes. Limnol. Oceanogr..

[66] Bach EM, Williams RJ, Hargreaves SK, Yang F, Hofmockel KS (2018). Greatest soil microbial diversity found in micro-habitats. Soil Biol Biochem.

[67] Franco M, De Mesel I, Demba Diallo M, Van Der Gucht K, Van Gansbeke D, Van Rijswijk P (2007). Effect of phytoplankton bloom deposition on benthic bacterial communities in two contrasting sediments in the southern North Sea. Aquat Microb Ecol..

[68] Bastida F, Eldridge DJ, García C, Kenny Png G, Bardgett RD, Delgado-Baquerizo M (2021). Soil microbial diversity–biomass relationships are driven by soil carbon content across global biomes. ISME J.

[69] Geyer KM, Barrett JE (2019). Unimodal productivity–diversity relationships among bacterial communities in a simple polar soil ecosystem. Environ Microbiol.

[70] Neumann A, Hass HC, Möbius J, Naderipour C (2019). Ballasted flocs capture pelagic primary production and alter the local sediment characteristics in the coastal German Bight (North Sea). Geosciences.

[71] Sharp CE, Brady AL, Sharp GH, Grasby SE, Stott MB, Dunfield PF (2014). Humboldt’s spa: microbial diversity is controlled by temperature in geothermal environments. ISME J.

[72] Zhou J, Deng Y, Shen L, Wen C, Yan Q, Ning D (2016). Temperature mediates continental-scale diversity of microbes in forest soils. Nat Commun.

[73] Delgado-Baquerizo M, Eldridge DJ (2019). Cross-biome drivers of soil bacterial alpha diversity on a worldwide scale. Ecosystems.

[74] Li J, Bates KA, Hoang KL, Hector TE, Knowles SCL, King KC (2023). Experimental temperatures shape host microbiome diversity and composition. Global Change Biol.

[75] Zaneveld JR, McMinds R, Vega Thurber R (2017). Stress and stability: applying the Anna Karenina principle to animal microbiomes. Nat Microbiol.

[76] Ahmed HI, Herrera M, Liew YJ, Aranda M (2019). Long-term temperature stress in the coral model aiptasia supports the “Anna Karenina Principle” for bacterial microbiomes. Front Microbiol..

[77] Bonthond G, Neu A, Bayer T, Krueger‐Hadfield SA, Künzel S, Weinberger F (2023). Non‐native hosts of an invasive seaweed holobiont have more stable microbial communities compared to native hosts in response to thermal stress. Ecol Evolut.

[78] He Q, Wang S, Hou W, Feng K, Li F, Hai W (2021). Temperature and microbial interactions drive the deterministic assembly processes in sediments of hot springs. Sci Total Environ.

[79] Kim M, Heo E, Kang H, Adams J (2013). Changes in soil bacterial community structure with increasing disturbance frequency. Microb Ecol.

[80] Amoroso RO, Pitcher CR, Rijnsdorp AD, McConnaughey RA, Parma AM, Suuronen P, et al. Bottom trawl fishing footprints on the world’s continental shelves. Proc Natl Acad Sci USA. 2018;115:E10275–82. 10.1073/pnas.1802379115 PMC6205437 30297399

[81] McLaverty C, Eigaard OR, Gislason H, Bastardie F, Brooks ME, Jonsson P (2020). Using large benthic macrofauna to refine and improve ecological indicators of bottom trawling disturbance. Ecol Indicat.

[82] Pitcher CR, Ellis N, Jennings S, Hiddink JG, Mazor T, Kaiser MJ (2017). Estimating the sustainability of towed fishing‐gear impacts on seabed habitats: a simple quantitative risk assessment method applicable to data‐limited fisheries. Methods Ecol Evol.

[83] Hiddink JG, Van De Velde SJ, McConnaughey RA, De Borger E, Tiano J, Kaiser MJ (2023). Quantifying the carbon benefits of ending bottom trawling. Nature.

[84] Seitzinger S, Harrison JA, Böhlke JK, Bouwman AF, Lowrance R, Peterson B (2006). Denitrificaiton across landscapes and waterscapes: a synthesis. Ecol Appl.

