# Supplementary material for: Benthic microbial biogeographic trends in the North Sea are shaped by an interplay of environmental drivers and bottom trawling effort
Source: ISME Commun. 2023 Dec 15;3:132. doi: 10.1038/s43705-023-00336-3 (PMC10724143; doi:10.1038/s43705-023-00336-3)
Supplement: Supplementary file 1 — Supplementary information [file 43705_2023_336_MOESM1_ESM.pdf]

Supporting information

Benthic microbial biogeographic trends in the North Sea are shaped by an interplay of environmental drivers and bottom trawling effort

Guido Bonthond[1,*], Jan Beermann[2], Lars Gutow[2], Andreas Neumann[3], Francisco Rafael Barboza[4], Andrea Desiderato[5,2], Vera Fofonova[2], Stephanie Helber[1], Sahar Khodami[6], Casper Kraan[7], Hermann Neumann[7], Sven Rohde[1], Peter J. Schupp[1,8]

[1] Institute for Chemistry and Biology of the Marine Environment (ICBM), Carl von Ossietzky University Oldenburg, Schleusenstrasse 1, 26382 Wilhelmshaven, Germany
[2] Alfred Wegener Institute Helmholtz Centre for Polar and Marine Research, Am Handelshafen 12, 27570 Bremerhaven, Germany
[3] Helmholtz Centre Hereon, Geesthacht 21502, Germany
[4] Estonian Marine Institute, University of Tartu, Mäealuse 14, 12618 Tallinn, Estonia
[5] Department of Invertebrate Zoology and Hydrobiology, University of Lodz, 90-136 Lodz, Poland
[6] Senckenberg am Meer Wilhelmshaven, German Centre for Marine Biodiversity Research, Südstrand 44, 26382, Wilhelmshaven, Germany
[7] Thünen Institute of Sea Fisheries, Herwigstraße 31, 27572 Bremerhaven, Germany
[8] Helmholtz Institute for Functional Marine Biodiversity at the University of Oldenburg (HIFMB), Ammerländer Heerstrasse 231, D-26129 Oldenburg

[*] Correspondence: guido.bonthond@uol.de

Table S1. Predicted KEGG Ortholog modules used in the analyses

Process	module	KO	symbol	name	EC
aerobic respiration	M00155	K02275	coxB	cytochrome c oxidase subunit II	7.1.1.9
		K02274	coxA	cytochrome c oxidase subunit I	
		K02276	coxC	cytochrome c oxidase subunit III	
		K15408	coxAC	cytochrome c oxidase subunit I III	
		K02277	coxD	cytochrome c oxidase subunit IV	
nitrification	M00528	K10944	pmoA-amoA	methane/ammonia monooxygenase subunit A	1.14.18.3 1.14.99.39
		K10945	pmoB-amoB	methane/ammonia monooxygenase subunit B	
		K10946	pmoC-amoC	methane/ammonia monooxygenase subunit C	
		K10535	hao	hydroxylamine dehydrogenase	1.7.2.6
dissimilatory nitrate reduction M00530.1	M00530	K02567	napA	nitrate reductase cytochrome	1.9.6.1
		K02568	napB	nitrate reductase cytochrome electron transfer subunit	
		K00370	narG	nitrate reductase/nitrite oxidoreductase alpha subunit	1.7.5.1 1.7.99.
		K00371	narH	nitrate reductase/nitrite oxidoreductase beta subunit	
		K00374	narI	nitrate reductase gamma subunit	
dissimilatory nitrite reduction M00530.2		K00362	nirB	nitrite reductase NADH large subunit	1.7.1.15
		K00363	nirD	nitrite reductase NADH small subunit	
		K03385	nrfA	nitrite reductase cytochrome c 552	1.7.2.2
		K15876	nrfH	cytochrome c nitrite reductase small subunit	
denitrification	M00529	K02567	napA	nitrate reductase cytochrome	1.9.6.1
		K02568	napB	nitrate reductase cytochrome electron transfer subunit	
		K00370	narG	nitrate reductase/nitrite oxidoreductase alpha subunit	1.7.5.1 1.7.99.
		K00371	narH	nitrate reductase/nitrite oxidoreductase beta subunit	
		K00374	narI	nitrate reductase gamma subunit	
		K00368	nirK	nitrite reductase NO forming	1.7.2.1
		K15864	nirS	nitrite reductase NO forming / hydroxylamine reductase	
		K04561	norB	nitric oxide reductase subunit B	1.7.2.5
		K02305	norC	nitric oxide reductase subunit C	
		K00376	nosZ	nitrous oxide reductase	1.7.2.4
dissimilatory sulfate reduction	M00596	K00958	sat	sulfate adenylyltransferase	2.7.7.4
		K00394	aprA	adenylylsulfate reductase subunit A	1.8.99.2
		K00395	aprB	denylylsulfate reductase subunit B	
		K11180	dsrA	dissimilatory sulfite reductase alpha subunit	1.8.99.5
		K11181	dsrB	dissimilatory sulfite reductase beta subunit	
Thiosulfate oxidation	M00595	K17222	soxA	L cysteine S thiosulfotransferase	2.8.5.2
		K17223	soxX	L cysteine S thiosulfotransferase	
		K17224	soxB	S sulfosulfanyl L cysteine sulfohydrolase	3.1.6.20
		K17225	soxC	sulfane dehydrogenase subunit SoxC	
		K17226	soxY	sulfur oxidizing protein SoxY	
		K17227	soxZ	sulfur oxidizing protein SoxZ	
methane oxidation	M00174	K10944	pmoA-amoA	methane/ammonia monooxygenase subunit A	1.14.18.3 1.14.99.39
		K10945	pmoB-amoB	methane/ammonia monooxygenase subunit B	
		K10946	pmoC-amoC	methane/ammonia monooxygenase subunit C	
		K16157	mmoX	methane monooxygenase component A alpha chain	1.14.13.25
		K16158	mmoY	methane monooxygenase component A beta chain	
		K16159	mmoZ	methane monooxygenase component A gamma chain	
		K16161	mmoC	methane monooxygenase component C	
		K16160	mmoB	methane monooxygenase regulatory protein B	
		K16162	mmoD	methane monooxygenase component D	
		K14028	mxaF	methanol dehydrogenase cytochrome c subunit 1	1.1.2.7
		K14029	mxaI	methanol dehydrogenase cytochrome c subunit 2	1.1.2.7
		K23995	xoxF	lanthanide dependent methanol dehydrogenase	1.1.2.10

Table S1 (continued). Predicted KEGG Ortholog modules used in the analyses

Process	module	KO	symbol	name	EC
		K00200	fwdA	formylmethanofuran dehydrogenase subunit A	
		K00201	fwdB	formylmethanofuran dehydrogenase subunit B	
		K00202	fwdC	formylmethanofuran dehydrogenase subunit C	1.2.7.12
		K00203	fwdD	formylmethanofuran dehydrogenase subunit D	
		K11261	fwdE	formylmethanofuran dehydrogenase subunit E	
		K00205	fwdF	4Fe-4S ferredoxin	
		K11260	fwdG	4Fe-4S ferredoxin	
		K00204	fwdH	4Fe-4S ferredoxin	
		K00672	ftr	formylmethanofuran--tetrahydromethanopterin N-formyltransferase	2.3.1.101
		K01499	mch	methenyltetrahydromethanopterin cyclohydrolase	3.5.4.27
		K00319	mtd	methylenetetrahydromethanopterin dehydrogenase	1.5.98.1
		K13942	hmd	5,10-methenyltetrahydromethanopterin hydrogenase	1.12.98.2
		K00320	mer	5,10-methylenetetrahydromethanopterin reductase	1.5.98.2
		K00577	mtrA	tetrahydromethanopterin S-methyltransferase subunit A	
		K00578	mtrB	tetrahydromethanopterin S-methyltransferase subunit B	
		K00579	mtrC	tetrahydromethanopterin S-methyltransferase subunit C	
		K00580	mtrD	tetrahydromethanopterin S-methyltransferase subunit D	2.1.1.86
		K00581	mtrE	tetrahydromethanopterin S-methyltransferase subunit E	
CO_2 reduction	M00567	K00582	mtrF	tetrahydromethanopterin S-methyltransferase subunit F	
		K00583	mtrG	tetrahydromethanopterin S-methyltransferase subunit G	
		K00584	mtrH	tetrahydromethanopterin S-methyltransferase subunit H	
		K00399	mcrA	methyl-coenzyme M reductase alpha subunit	
		K00401	mcrB	methyl-coenzyme M reductase beta subunit	2.8.4.1
		K00402	mcrG	methyl-coenzyme M reductase gamma subunit	
		K08264	hdrD	heterodisulfide reductase subunit D	1.8.98.1
		K08265	hdrE	heterodisulfide reductase subunit E	
		K22480	hdrA1	heterodisulfide reductase subunit A1	
		K22481	hdrB1	heterodisulfide reductase subunit B1	
		K22482	hdrC1	heterodisulfide reductase subunit C1	
		K03388	hdrA2	heterodisulfide reductase subunit A2	
		K03389	hdrB2	heterodisulfide reductase subunit B2	1.8.7.3<br>1.8.98.4<br>1.8.98.5<br>1.8.98.6
		K03390	hdrC2	heterodisulfide reductase subunit C2	
		K14127	mvhD	F420-non-reducing hydrogenase iron-sulfur subunit	
		K14126	mvhA	F420-non-reducing hydrogenase large subunit	
		K14128	mvhG	F420-non-reducing hydrogenase small subunit	
		K22516	fdhA	formate dehydrogenase (coenzyme F420) alpha subunit	
		K00125	fdhB	formate dehydrogenase (coenzyme F420) beta subunit	

Abbreviations: KEGG Ortholog (KO), Enzyme Commission number (EC)

Table S2. Dominant genera

phylum	class	order	family	genus	dominance[1]
Proteobacteria	γ-proteobacteria	Steroidobacterales	Woeseiaceae	*Woeseia*	95
Proteobacteria	δ-proteobacteria	Myxococcales	Sandaracinaceae	*NA*	84
Proteobacteria	α-proteobacteria	Tistrellales	Geminicoccaceae	*NA*	31
Actinobacteria	Acidimicrobiia	Actinomarinales	uncultured	*NA*	31
Actinobacteria	Acidimicrobiia	Microtrichales	Microtrichaceae	Sva0996 marine group	13
Planctomycetes	Planctomycetacia	Pirellulales	Pirellulaceae	*Rhodopirellula*	11
Planctomycetes	Planctomycetacia	Pirellulales	Pirellulaceae	*Blastopirellula*	10
Proteobacteria	γ-proteobacteria	*NA*	*NA*	*NA*	8
Bacteroidetes	Bacteroidia	Flavobacteriales	Flavobacteriaceae	*Eudoraea*	8
Nitrospirae	Nitrospira	Nitrospirales	Nitrospiraceae	*Nitrospira*	6
Proteobacteria	γ-proteobacteria	BD7-8	BD7-8	BD7-8	5
Actinobacteria	Acidimicrobiia	Microtrichales	Microtrichaceae	*NA*	5
Proteobacteria	γ-proteobacteria	Ectothiorhodospirales	Ectothiorhodospiraceae	*Thiogranum*	4
Verrucomicrobia	Verrucomicrobiae	Verrucomicrobiales	DEV007	DEV007	4
Proteobacteria	γ-proteobacteria	Alteromonadales	Shewanellaceae	*Shewanella*	3
Proteobacteria	γ-proteobacteria	Cellvibrionales	Halieaceae	*Halioglobus*	3
Proteobacteria	γ-proteobacteria	γ-proteobacteria *IS*	*NA*	*NA*	3
Proteobacteria	δ-proteobacteria	Desulfobacterales	Desulfobacteraceae	Sva0081 sedimentgroup	3
Proteobacteria	γ-proteobacteria	D90	D90	D90	2
Kiritimatiellaeota	Kiritimatiellae	Kiritimatiellales	Kiritimatiellaceae	R76-B128	2
Proteobacteria	γ-proteobacteria	Alteromonadales	Psychromonadaceae	*Psychromonas*	1
Proteobacteria	γ-proteobacteria	B2M28	B2M28	B2M28	1
Proteobacteria	δ-proteobacteria	NB1-j	NB1-j	NB1-j	1
Proteobacteria	α-proteobacteria	Thalassobaculales	*NA*	*NA*	1
Entotheonellaeota	Entotheonellia	Entotheonellales	Entotheonellaceae	*Candidatus* Entotheonella	1
Bacteroidetes	Bacteroidia	Cytophagales	Cyclobacteriaceae	*NA*	1
Bacteroidetes	Bacteroidia	Flavobacteriales	Flavobacteriaceae	*Lutimonas*	1
Acidobacteria	Thermoanaerobaculia	Thermoanaerobaculales	Thermoanaerobaculaceae	Subgroup 10	1

Abbreviations: Incertae Sedis (IS)

[1]number of samples in which taxon was dominant

Table S3. PERMANOVA and mGLM output

	PERMANOVA							mGLM		
	variable	df	SumOfSqs	R^2	F value	Pr(>F)[1]		Deviance	significant responses +	significant responses −
OTU composition	LSD	1	264.6097	0.024415	9.618925	0.0001	***	337321	8591	20
	MEM1	1	85.36657	0.007877	3.103191	0.0001	***	138779	1196	623
	MEM2	1	34.69398	0.003201	1.261174	0.0001	***	99658	807	713
	SAR	1	50.22911	0.004635	1.825897	0.0001	***	160742	782	949
	temperature	1	35.72422	0.003296	1.298624	0.0001	***	114381	349	820
	median grain size	1	401.58	0.037053	14.59798	0.0001	***	471364	2682	4870
	logit TOM	1	62.4307	0.00576	2.269441	0.0089	**	139156	516	1442
	√ mud content	1	74.52633	0.006876	2.709134	0.0001	***	209273	1538	2161
	shear stress	1	46.97246	0.004334	1.707513	0.0018	**	123800	617	960
	Residuals	329	9050.554	0.83507						
	Total	338	10838.07							
genus composition	LSD	1	123.7386	0.020919	8.24162	0.0001	***	51214	796	0
	MEM1	1	43.47662	0.00735	2.895765	0.0001	***	7092	178	85
	MEM2	1	19.16939	0.003241	1.276779	0.0001	***	2495	25	61
	SAR	1	27.104	0.004582	1.805265	0.0001	***	4433	78	93
	temperature	1	18.5324	0.003133	1.234352	0.0001	***	2917	31	84
	median grain size	1	242.8744	0.04106	16.17667	0.0001	***	61021	242	369
	logit TOM	1	28.83238	0.004874	1.920384	0.0561		32181	96	113
	√ mud content	1	41.73871	0.007056	2.780011	0.0001	***	8223	228	115
	shear stress	1	21.27785	0.003597	1.417213	0.6313		2995	42	52
	Residuals	329	4939.561	0.835066						
	Total	338	5915.175							
Predicted KO composition	LSD	1	541.6887	0.02187	8.122905	0.0001	***	2203066	6596	1
	MEM1	1	157.5702	0.006362	2.362847	0.0001	***	602716	2306	1268
	MEM2	1	71.07529	0.00287	1.065811	0.0001	***	30189	344	402
	SAR	1	104.0757	0.004202	1.560669	0.0001	***	153528	882	864
	temperature	1	88.76827	0.003584	1.331127	0.0001	***	179928	750	938
	median grain size	1	559.8909	0.022605	8.395857	0.0001	***	2282055	2118	3158
	logit TOM	1	92.77844	0.003746	1.391261	0.0001	***	525996	1505	1204
	√ mud content	1	178.303	0.007199	2.673747	0.0001	***	394540	2768	1123
	shear stress	1	77.42259	0.003126	1.160992	0.0002	***	112655	431	947
	Residuals	329	21939.88	0.885806						
	Total	338	24768.26							

Abbreviations: multivariate Generalized Linear Model (mGLM), Log-transformed Sequencing Depth (LSD), Moran Eigenvector Map (MEM), Total Organic Matter (TOM), Swept Area Ratio (SAR).
[1]Significance codes from PERMANOVA: < 0.001 (***), < 0.01 (**), < 0.05 (*)

Table S4. AIC_c tables

<table>
<tr><th rowspan="3">response[1]</th><th colspan="8">predictor[2]</th><th colspan="5">model performance statistics</th></tr>
<tr><th colspan="2">LSD[3]</th><th rowspan="2">grain</th><th rowspan="2">TOM</th><th rowspan="2">shear</th><th rowspan="2">mud</th><th rowspan="2">temp</th><th rowspan="2">SAR</th><th rowspan="2">df</th><th rowspan="2">logL</th><th rowspan="2">AICc</th><th rowspan="2">ΔAICc</th><th rowspan="2">AICcw</th></tr>
<tr><th>x</th><th>y</th></tr>
<tr><td>OTU diversity[4]</td><td colspan="2">+</td><td>+</td><td></td><td>+</td><td>+</td><td></td><td>+</td><td>13</td><td>-1870.2</td><td>3767.5</td><td>0.000</td><td>0.342</td></tr>
<tr><td></td><td colspan="2"></td><td>+</td><td></td><td>+</td><td>+</td><td></td><td>+</td><td>12</td><td>-1871.4</td><td>3767.8</td><td>0.275</td><td>0.298</td></tr>
<tr><td></td><td colspan="2">+</td><td>+</td><td>+</td><td>+</td><td>+</td><td></td><td>+</td><td>15</td><td>-1869.8</td><td>3771.0</td><td>3.521</td><td>0.059</td></tr>
<tr><td></td><td colspan="2"></td><td>+</td><td>+</td><td>+</td><td>+</td><td></td><td>+</td><td>14</td><td>-1871.1</td><td>3771.4</td><td>3.919</td><td>0.048</td></tr>
<tr><td>Genus level diversity</td><td colspan="2"></td><td>+</td><td></td><td>+</td><td>+</td><td></td><td>+</td><td>12</td><td>-1276.7</td><td>2578.3</td><td>0.000</td><td>0.213</td></tr>
<tr><td></td><td colspan="2">+</td><td>+</td><td></td><td>+</td><td>+</td><td></td><td>+</td><td>13</td><td>-1276.0</td><td>2579.1</td><td>0.742</td><td>0.147</td></tr>
<tr><td></td><td colspan="2"></td><td>+</td><td></td><td></td><td>+</td><td>+</td><td>+</td><td>12</td><td>-1277.4</td><td>2579.7</td><td>1.355</td><td>0.108</td></tr>
<tr><td></td><td colspan="2">+</td><td>+</td><td></td><td></td><td>+</td><td>+</td><td>+</td><td>13</td><td>-1276.8</td><td>2580.6</td><td>2.285</td><td>0.068</td></tr>
<tr><td></td><td colspan="2"></td><td>+</td><td></td><td>+</td><td>+</td><td>+</td><td>+</td><td>14</td><td>-1275.7</td><td>2580.7</td><td>2.348</td><td>0.066</td></tr>
<tr><td></td><td colspan="2">+</td><td>+</td><td></td><td>+</td><td>+</td><td>+</td><td>+</td><td>15</td><td>-1275.0</td><td>2581.5</td><td>3.203</td><td>0.043</td></tr>
<tr><td></td><td colspan="2"></td><td>+</td><td></td><td></td><td>+</td><td>+</td><td></td><td>10</td><td>-1280.7</td><td>2582.1</td><td>3.755</td><td>0.033</td></tr>
<tr><td>Predicted KO diversity</td><td colspan="2"></td><td>+</td><td>+</td><td></td><td>+</td><td>+</td><td>+</td><td>14</td><td>-1711.7</td><td>3452.8</td><td>0.000</td><td>0.206</td></tr>
<tr><td></td><td colspan="2"></td><td>+</td><td></td><td></td><td>+</td><td>+</td><td></td><td>10</td><td>-1716.4</td><td>3453.6</td><td>0.783</td><td>0.139</td></tr>
<tr><td></td><td colspan="2"></td><td>+</td><td></td><td></td><td>+</td><td>+</td><td>+</td><td>12</td><td>-1714.4</td><td>3453.8</td><td>0.994</td><td>0.125</td></tr>
<tr><td></td><td colspan="2">+</td><td>+</td><td>+</td><td></td><td>+</td><td>+</td><td>+</td><td>15</td><td>-1711.7</td><td>3455.0</td><td>2.187</td><td>0.069</td></tr>
<tr><td></td><td colspan="2"></td><td>+</td><td>+</td><td>+</td><td>+</td><td>+</td><td></td><td>14</td><td>-1712.9</td><td>3455.1</td><td>2.307</td><td>0.065</td></tr>
<tr><td></td><td colspan="2"></td><td>+</td><td>+</td><td></td><td>+</td><td>+</td><td></td><td>12</td><td>-1715.1</td><td>3455.2</td><td>2.419</td><td>0.061</td></tr>
<tr><td></td><td colspan="2">+</td><td>+</td><td></td><td></td><td>+</td><td>+</td><td></td><td>11</td><td>-1716.4</td><td>3455.7</td><td>2.903</td><td>0.048</td></tr>
<tr><td></td><td colspan="2">+</td><td>+</td><td></td><td></td><td>+</td><td>+</td><td>+</td><td>13</td><td>-1714.4</td><td>3455.9</td><td>3.149</td><td>0.043</td></tr>
<tr><td>OTU beta diversity</td><td>+</td><td>+</td><td>+</td><td></td><td></td><td>+</td><td></td><td></td><td>10</td><td>-837.8</td><td>1696.4</td><td>0.000</td><td>0.384</td></tr>
<tr><td></td><td>+</td><td>+</td><td>+</td><td>+</td><td></td><td>+</td><td></td><td></td><td>12</td><td>-836.5</td><td>1698.1</td><td>1.698</td><td>0.164</td></tr>
<tr><td></td><td>+</td><td>+</td><td>+</td><td></td><td>+</td><td>+</td><td></td><td></td><td>12</td><td>-836.7</td><td>1698.6</td><td>2.208</td><td>0.127</td></tr>
<tr><td></td><td>+</td><td>+</td><td>+</td><td></td><td></td><td>+</td><td></td><td>+</td><td>12</td><td>-837.2</td><td>1699.6</td><td>3.210</td><td>0.077</td></tr>
<tr><td>genus beta diversity</td><td>+</td><td></td><td></td><td>+</td><td>+</td><td>+</td><td></td><td></td><td>10</td><td>-649.5</td><td>1319.8</td><td>0.000</td><td>0.324</td></tr>
<tr><td></td><td>+</td><td></td><td>+</td><td>+</td><td>+</td><td>+</td><td></td><td></td><td>12</td><td>-648.0</td><td>1321.1</td><td>1.244</td><td>0.174</td></tr>
<tr><td></td><td></td><td></td><td></td><td>+</td><td>+</td><td></td><td></td><td></td><td>8</td><td>-653.0</td><td>1322.5</td><td>2.685</td><td>0.085</td></tr>
<tr><td></td><td>+</td><td>+</td><td></td><td>+</td><td>+</td><td>+</td><td></td><td></td><td>12</td><td>-649.0</td><td>1323.2</td><td>3.432</td><td>0.058</td></tr>
</table>

Table S4 (continued). AIC_c tables

response[1]	predictor[2]								model performance statistics				
	LSD[3]		grain	TOM	shear	mud	temp	SAR	df	logL	AICc	ΔAICc	AICcw
	x	y											
Predicted KO beta diversity			+						6	-1217.2	2446.7	0.000	0.073
		+	+						7	-1216.2	2446.7	0.086	0.070
		+	+	+					7	-1216.2	2446.8	0.142	0.068
			+	+					9	-1214.3	2447.3	0.638	0.053
				+					8	-1215.5	2447.5	0.840	0.048
		+		+					6	-1217.6	2447.6	0.940	0.046
		+	+			+			8	-1215.8	2448.1	1.452	0.035
			+			+			8	-1215.8	2448.1	1.460	0.035
	+		+						5	-1219	2448.2	1.508	0.034
	+	+	+						7	-1216.9	2448.2	1.589	0.033
									7	-1216.9	2448.3	1.631	0.032
	+	+	+	+					9	-1214.8	2448.3	1.681	0.032
		+							8	-1216	2448.4	1.766	0.030
		+	+	+		+			10	-1214	2448.9	2.243	0.024
	+		+	+					6	-1218.3	2448.9	2.253	0.024
		+	+					+	4	-1220.4	2449	2.307	0.023
	+			+					6	-1218.4	2449	2.366	0.022
			+					+	7	-1217.3	2449.1	2.441	0.022
	+	+		+					9	-1215.3	2449.2	2.521	0.021
			+	+		+			11	-1213.1	2449.2	2.568	0.020
								+	7	-1217.4	2449.2	2.576	0.020
		+	+		+				8	-1216.4	2449.2	2.583	0.020
			+		+				8	-1216.4	2449.3	2.672	0.019
		+						+	9	-1215.3	2449.3	2.676	0.019
	+	+	+			+			8	-1216.6	2449.7	3.047	0.016
	+		+			+			10	-1214.5	2449.7	3.061	0.016
Predicted aerobic respiration	+				+			+	9	535.3	-1052.0	0.000	0.433
	+				+		+	+	11	536.6	-1050.5	1.562	0.198
	+				+	+		+	11	536.1	-1049.3	2.681	0.113
Predicted nitrification	+		+	+	+			+	13	-344.6	716.3	0.000	0.299
	+		+		+			+	11	-346.9	716.5	0.243	0.265
	+		+		+	+		+	13	-345.4	718.0	1.684	0.129
	+		+	+	+	+		+	15	-343.5	718.4	2.139	0.103
	+		+	+	+		+	+	15	-344.4	720.2	3.943	0.042
Predicted dissimilatory nitrate reduction	+		+	+	+			+	13	-162.6	352.3	0.000	0.607
	+		+	+	+		+	+	15	-161.5	354.5	2.142	0.208
	+		+	+	+	+		+	15	-162.2	355.9	3.602	0.100

Table S4 (continued). AIC_c tables

response[1]	predictor[2]							model performance statistics				
	LSD[3] x y	grain	TOM	shear	mud	temp	SAR	df	logL	AICc	ΔAICc	AICcw
Predicted dissimilatory nitrite reduction	+		+		+			9	113.9	-209.2	0.000	0.173
	+	+						7	111.0	-207.6	1.537	0.080
	+		+	+	+			11	115.1	-207.3	1.826	0.069
	+				+			7	110.7	-207.1	2.023	0.063
	+	+	+		+			11	114.8	-206.8	2.345	0.054
	+	+			+			9	112.5	-206.5	2.636	0.046
	+	+		+				9	112.5	-206.4	2.800	0.043
	+		+		+	+		11	114.3	-205.8	3.313	0.033
	+	+					+	9	112.1	-205.6	3.536	0.030
	+		+		+		+	11	114.2	-205.6	3.599	0.029
	+	+	+					9	112.0	-205.5	3.631	0.028
	+			+	+			9	112.0	-205.4	3.778	0.026
	+			+				7	109.8	-205.3	3.884	0.025
Predicted denitrification	+	+	+				+	11	-31.81	86.42	0.000	0.394
	+	+	+			+	+	13	-29.69	86.50	0.084	0.378
	+	+	+	+		+	+	15	-29.15	89.78	3.364	0.073
Predicted sulfate reduction	+	+		+			+	11	31.06	-39.32	0.000	0.458
	+	+		+	+		+	13	31.97	-36.82	2.501	0.131
	+	+					+	9	27.61	-36.68	2.645	0.122
	+	+	+	+			+	13	31.70	-36.27	3.050	0.100
Predicted thiosulfate oxidation	+	+	+	+			+	13	-2.841	32.80	0.000	0.201
	+	+	+				+	11	-5.314	33.43	0.632	0.147
	+	+			+		+	11	-5.565	33.94	1.136	0.114
	+	+	+	+		+	+	15	-1.300	34.09	1.283	0.106
	+	+	+		+		+	13	-3.622	34.36	1.562	0.092
	+	+					+	9	-8.153	34.85	2.050	0.072
	+	+	+	+	+		+	15	-1.816	35.12	2.316	0.063
	+	+	+			+	+	13	-4.265	35.65	2.846	0.049
	+	+	+	+	+	+	+	17	-0.441	36.79	3.985	0.027
Predicted methane oxidation	+	+		+			+	11	-255.9	534.6	0.000	0.449
	+	+		+	+		+	13	-254.5	536.1	1.566	0.205
	+	+	+	+			+	13	-255.2	537.5	2.923	0.104
	+	+		+		+	+	13	-255.2	537.6	3.007	0.100
Predicted CO_2 reduction	+	+	+					9	27.659	-36.8	0.000	0.378
	+	+	+	+				11	28.912	-35.0	1.756	0.157
	+	+	+				+	11	28.346	-33.9	2.888	0.089
	+	+	+		+			11	27.890	-33.0	3.800	0.057

Abbreviations: degrees of freedom (df), log likelihood score (logL), corrected Akaike's information criterion (AICc), difference in AICc relative to the best model (ΔAICc), AICc weight (AICcw)

[1] Only models with a ΔAICc ≤ 4 are shown

[2] Predictors retained in the models are indicated with (+). The intercept, random effect and covariance structure were included in all models considered in the selection procedure

[3] For beta diversity responses, the sequencing depths of both samples were included as parametric predictors (seq_depth.x and seq_depth.y) without log transformation

[4] Diversity is measured in effective numbers of OTUs, genera or predicted KOs

Table S5. GAMM output

response	predictor	variable type	edf	F-value	p-value	significance	RI
OTU diversity[1]	LSD	parametric	1.000	2.403	0.122		0.54
	grain	Smoother	2.784	16.713	< 0.001	***	1.00
	shear	smoother	1.000	9.776	0.002	**	1.00
	sqrt(mud)	smoother	2.498	5.984	0.001	**	1.00
	SAR	smoother	2.672	10.013	< 0.001	***	1.00
	station	random intercept	81.286	1.655	< 0.001	***	*NA*
genus level diversity	grain	smoother	2.52	8.867	< 0.001	***	1.00
	shear	smoother	1.00	9.553	0.002	**	0.69
	sqrt(mud)	smoother	2.35	8.808	< 0.001	***	1.00
	SAR	smoother	1.02	8.870	0.003	**	1.00
	station	random intercept	65.26	0.953	< 0.001	***	*NA*
predicted KO diversity	grain	smoother	1.00	62.66	< 0.001	***	1.00
	logit(TOM)	smoother	2.32	3.904	0.033	*	0.53
	sqrt(mud)	smoother	2.37	6.796	0.002	**	1.00
	temperature	smoother	1.23	5.052	0.012	*	0.59
	SAR	smoother	1.70	7.279	0.018	*	1.00
	station	random intercept	69.01	1.123	< 0.001	***	*NA*
OTU beta diversity	seq_depth.x	parametric	1.000	289.290	< 0.001	***	1.00
	seq_depth.y	parametric	1.000	285.992	< 0.001	***	1.00
	grain	smoother	2.610	13.167	< 0.001	***	1.00
	sqrt(mud)	smoother	1.897	9.484	< 0.001	***	1.00
	station	random intercept	37.839	0.699	< 0.001	***	*NA*
genus beta diversity	seq_depth.x	parametric	1.000	75.514	< 0.001	***	1.00
	seq_depth.y	parametric	1.000	101.963	< 0.001	***	1.00
	grain	smoother	1.000	7.020	0.009	**	0.87
	sqrt(mud)	smoother	2.326	13.150	< 0.001	***	1.00
	station	random intercept	46.636	1.025	< 0.001	***	*NA*
Predicted KO beta diversity	grain	parametric	2.206	4.523	0.010	*	0.72
	station	random intercept	26.862	0.403	0.018	*	*NA*
Predicted aerobic respiration log(M00155)	lsd	parametric	1.000	51016.485	< 0.001	***	1.00
	shear	smoother	1.977	41.889	< 0.001	***	1.00
	SAR	smoother	2.180	38.040	< 0.001	***	1.00
	station	random intercept	87.611	1.625	< 0.001	***	*NA*
Predicted denitrification log(M00528)	LSD	parametric	1.000	1837.099	< 0.001	***	1.00
	grain	smoother	1.579	168.739	< 0.001	***	1.00
	logit(TOM)	smoother	2.818	5.944	< 0.001	***	1.00
	SAR	smoother	1.000	41.166	< 0.001	***	1.00
	station	random intercept	90.325	2.136	< 0.001	***	*NA*
Predicted dissimilatory nitrate reduction log(M00530.1)	LSD	parametric	1.000	971.832	< 0.001	***	1.00
	grain	smoother	1.385	94.261	< 0.001	***	1.00
	logit(TOM)	smoother	2.735	6.531	0.006	**	1.00
	shear	smoother	2.543	16.621	< 0.001	***	1.00
	SAR	smoother	2.911	18.889	< 0.001	***	1.00
	station	random intercept	94.147	2.435	< 0.001	***	*NA*
Predicted dissimilatory nitrite reduction log(M00530.2)	LSD	parametric	1.000	4593.185	< 0.001	***	1.00
	logit(TOM)	smoother	2.699	4.810	0.024	*	0.55
	sqrt(mud)	smoother	1.906	6.800	0.001	**	0.71
	station	random intercept	39.394	0.386	0.005	**	*NA*
Predicted nitrification log1p(M00528)	LSD	parametric	1.000	293.857	< 0.001	***	1.00
	grain	smoother	2.440	36.269	< 0.001	***	1.00
	logit(TOM)	smoother	2.351	2.822	0.039	*	0.59
	shear	smoother	2.730	23.898	< 0.001	***	1.00
	SAR	smoother	2.217	5.661	0.005	**	1.00
	station	random intercept	83.944	1.862	< 0.001	***	*NA*

Table S5 (continued). GAMM output

response	predictor	variable type	edf	F-value	p-value	significance	RI
Predicted dissimilatory sulfate reduction log(M00596)	LSD	parametric	1.000	2296.206	< 0.001	***	1.00
	grain	smoother	2.899	240.355	< 0.001	***	1.00
	shear	smoother	1.000	8.391	0.004	**	0.85
	SAR	smoother	1.989	15.627	< 0.001	***	1.00
	station	random intercept	80.456	1.415	< 0.001	***	*NA*
Predicted thiosulfate oxidation log(M00595)	LSD	parametric	1.000	1898.001	< 0.001	***	1.00
	grain	smoother	2.806	65.150	< 0.001	***	1.00
	logit(TOM)	smoother	2.325	4.704	0.008	**	0.79
	shear	smoother	2.653	4.141	0.046	*	0.46
	SAR	smoother	2.405	8.144	< 0.001	***	1.00
	station	random intercept	84.342	1.835	< 0.001	***	*NA*
Predicted methane oxidation log(M00174)	LSD	parametric	1.000	470.313	< 0.001	***	1.00
	grain	smoother	2.344	23.360	< 0.001	***	1.00
	shear	smoother	2.645	17.424	< 0.001	***	1.00
	SAR	smoother	2.351	7.119	< 0.001	***	1.00
	station	random intercept	96.100	2.709	< 0.001	***	*NA*
Predicted CO_2 reduction log(M00174)	LSD	parametric	1.000	2384.628	< 0.001	***	1.00
	grain	smoother	2.845	194.787	< 0.001	***	1.00
	logit(TOM)	smoother	2.522	4.577	0.007	**	1.00
	station	random intercept	86.656	1.825	< 0.001	***	*NA*

Abbreviations: KEGG Ortholog (KO), Relative Importance (RI), Log transformed sequencing depth (LSD), Total Organic Matter (TOM)

[1] Diversity is measured in effective numbers of OTUs, genera or predicted KOs

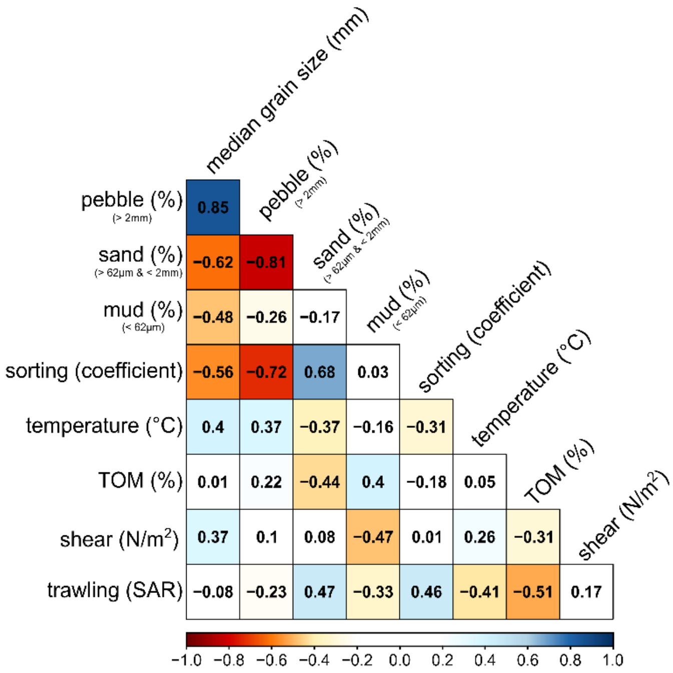

Figure S1. Correlation matrix based on spearman rank coefficients

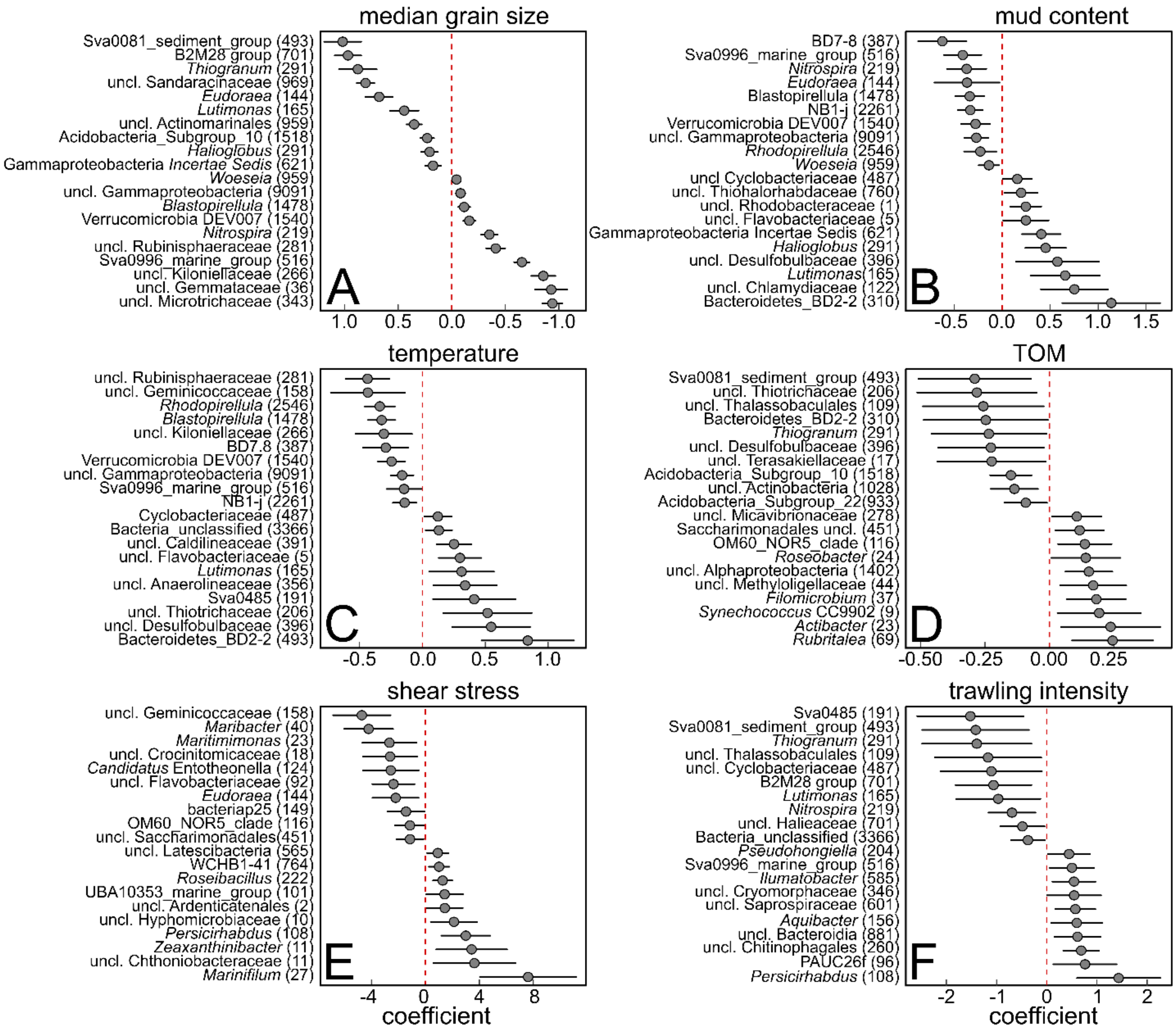

Figure S2. Differentially abundant genera associated with all included predictors of interest obtained from the multivariate GLMs. Only the most abundant 15 genera are shown. The number of OTUs within each genus or genus level grouping is indicated within brackets. Coefficients are in the scale of the link function.
